# Accurately Estimating Correlations Between Demographic Parameters: A Response to Riecke Et al. (2024)

**DOI:** 10.1002/ece3.71004

**Published:** 2025-02-24

**Authors:** Cody E. Deane, Lindsay G. Carlson, Curry J. Cunningham, Pat Doak, Knut Kielland, Greg A. Breed

**Affiliations:** ^1^ University of Alaska Fairbanks Department of Biology and Wildlife Fairbanks Alaska USA; ^2^ University of Saskatchewan Department of Biology Saskatoon Saskatchewan Canada; ^3^ University of Alaska Fairbanks College of Fisheries and Ocean Sciences Juneau Alaska USA; ^4^ University of Alaska Fairbanks Institute of Arctic Biology Fairbanks Alaska USA

**Keywords:** Bayesian analysis, capture‐mark‐recapture models, hierarchical models, multivariate normal distribution, random effects

## Abstract

Correlations between annual recovery and survival probabilities estimated from tag‐recovery data have been used to quantify the demographic response of exploited populations to harvest. Deane et al. (2023) evaluated the bias and certainty of correlation parameters between recovery and survival probabilities estimated as random effects drawn from bivariate normal distributions relative to different prior distributions and sample size combinations. Riecke et al. (2024) observed that we incorrectly parameterized a precision matrix with Gamma priors and suggested using a Gamma(1,1) prior distribution for the standard deviations as an alternative. Riecke et al. (2024) provided results from tag‐recovery models that estimate mortality hazard rates after fitting these models to tag‐recovery datasets with large sample sizes. Here, we fit tag‐recovery models to the data we previously simulated (Deane et al. 2023) while using Gamma(1,1) as the prior distribution for standard deviations while parameterizing these models to estimate recovery and survival in discrete time or to estimate cause‐specific mortality as hazard rates. We compare our new results to previous results obtained while using Uniform(0,5) prior distribution for the standard deviations. When sample sizes were large, correlation estimates obtained with either prior distribution provided similarly reliable parameter recovery and inference, replicating results of Riecke et al. (2024). With smaller sample sizes similar to those available for most duck populations in North America, correlations estimated with either prior distribution were uncertain and ambiguous. With decreasing sample sizes, annual survival was estimated with increasing uncertainty when compared to annual recovery, likely contributing to the poor ability to estimate correlation. Consistent with the original interpretation of Deane et al. (2023) and previous literature, we found correlations were often estimated with high uncertainty such that the sign (+ or –) may be the only attribute of these parameters that can be reliably interpreted.

## Graphical abstract

Correlations between annual recovery and survival probabilities estimated as random effects drawn from multivariate normal distributions have been used to quantify the demographic response of exploited populations to harvest. We fit tag‐recovery models to simulated datasets using Uniform(0,5) and Gamma(1,1) as prior distributions for standard deviations. When sample sizes were large, correlation estimates obtained with either prior distribution provided similarly reliable parameter recovery, and when sample sizes were more modest, correlation estimates using either prior distribution provided mostly unreliable inference.

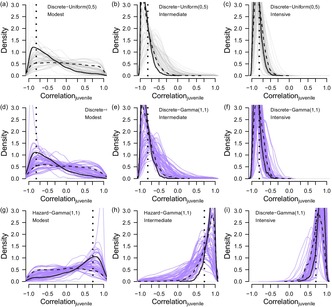

## 1 Introduction

Correlations between demographic parameters estimated as random effects drawn from bivariate or multivariate normal distributions can be a useful method for quantifying ecological relationships (Link and Barker 2005). In the case of harvested populations for which tag‐recovery data are available, correlation between annual harvest recovery, and survival probabilities has been estimated to assess the survival response of harvested populations to human exploitation (Sedinger et al. 2010, Iverson et al. 2014, Arnold et al. 2016, Bartzen and Dufour 2017). Tag‐recovery datasets are advantageous in that long‐term tagging operations have been maintained for decades, and hunters contribute to monitoring by reporting recovered tags to agencies like the U.S. Geological Survey Bird Banding Lab (Arnold et al. 2018). A disadvantage of tag‐recovery datasets like those available for many duck populations is that only a small portion of each cohort is recovered. As such, there is often substantial uncertainty about the timing of unobserved mortality, and consequently, about the certainty of annual survival estimates. As the uncertainty of survival estimates increases, an expected outcome of estimating correlation between survival and indexes of harvest (e.g., harvest recovery probabilities) is increasingly uncertain estimates of correlation that become closer to 0.

Deane et al. (2023) evaluated the bias and certainty of correlations between annual harvest recovery and survival probabilities (*ρ*) when these parameters were estimated as random effects drawn from bivariate normal distributions. Riecke et al. (2024) observed an error in our parameterization of a precision matrix that applied Gamma prior distributions to the off‐diagonal positions (Deane et al. 2023; Equation 7). We included this parameterization to identify a prior formulation that could be applied to precision matrices instead of variance–covariance matrices. As an alternative, Riecke et al. (2024) demonstrate that a Gamma(1,1) prior distribution can be applied to the standard deviations of random effects (*σ*) when random effects are drawn from bivariate normal distributions. This prior distribution for the standard deviations resulted in the same estimates of correlation as a Uniform(0,5) prior distribution when fitting tag‐recovery models to simulated datasets with large sample sizes (Riecke et al. 2024). Riecke et al. (2024) estimate harvest and unobserved mortality as hazard rates (Ergon et al. 2018) in place of estimating annual harvest and survival as discrete time parameters per Deane et al. (2023). Hazard‐rate models are advantageous in that they model mortality by treating cause‐specific mortality as a continuous process rather than a discrete process (Ergon et al. 2018).

Here, we fit tag‐recovery models that estimate recovery and survival in discrete time and tag‐recovery models that estimate recovery and unobserved mortality as hazard rates to simulated datasets with modest, intermediate, and large sample sizes from Deane et al. (2023). We use Gamma(1,1) as the prior distribution for the standard deviations per Riecke et al. (2024) for discrete‐time and hazard‐rate models. We compare our new parameter estimates with our previous estimates when we used Uniform(0,5) as the prior distribution for standard deviations (Deane et al. 2023). In addition, we note a few areas for additional improvement when implementing tag‐recovery models and presenting correlation estimates. The goal of our response is to integrate the parameterization suggested by Riecke et al. (2024) into our previous analysis with both discrete‐time models and hazard‐rate models while using sample sizes typically available for waterfowl populations in North America.

## 2 Methods

To update our results while using Gamma(1,1) prior distribution for the standard deviations, we fit tag‐recovery models to simulated data from Deane et al. (2023). The power analysis of Deane et al. (2023) focused on how different combinations of sampling intensity and priors used for Bayesian parameter estimation capture the truth of a population (Deane et al. 2023, Figure 2). Deane et al. (2023) simulated a population comprised of 9 million individuals whose encounter history began in the hatch year (HY) or juvenile age class (250,000 new individuals per year) and 14.4 million individuals whose encounter history began in the after‐hatch year (AHY) or adult age class (400,000 new individuals per year); the key attribute of this simulated population was a strongly negative correlation between age‐specific recovery and survival probabilities (about −0.8). We obtained realized tag‐recovery datasets from the population we simulated by randomly sampling capture histories according to different monitoring scenarios (4 scenarios × 50 data realizations per scenario): (1) modest sampling in which 250 juveniles and 800 adults are tagged annually; (2) intermediate sampling with 2000 individuals of each age class tagged annually; and (3) an intensive scenario in which 10,000 individuals of each age class are tagged annually (Deane et al. 2023). Here, we do not use data from an additional scenario with modest and episodic sampling because the results from this scenario differed little from the other modest scenario (Deane et al. 2023). In summary, after fitting models to realized tag‐recovery datasets, we compared posterior estimates of correlation to the known values of correlation for the population we simulated (Deane et al. 2023).

We fit multinomial tag‐recovery models to realized tag‐recovery datasets for two age classes, juveniles (HY) and adults (AHY). In these models, the probability of observed tag‐recovery data organized in the *m*‐array format (**M**_*age,y*,1:*Y* + 1_) are a function of age‐specific annual harvest recovery probabilities (*f*) and survival (*S*) probabilities (Brownie et al. 1985). We estimated the probability of observed tag‐recovery data (**M**_*age,y*,1:*Y* + 1_) with multinomial distributions parameterized by success probabilities specific to age class and total individuals released annually (**R**_*age,y*_) or annual cohort size (Equation 1)

(1) $$\begin{array}{c} M_{\mathrm{HY},y,1:Y+1}\sim \text{Multinomial}\left(\alpha_{y,1:Y},R_{\mathrm{HY},y,1:Y}\right)\\ M_{\mathrm{AHY},y,1:Y+1}\sim \text{Multinomial}\left(\beta_{y,1:Y},R_{\mathrm{AHY},y,1:Y}\right) \end{array}$$

We defined age‐specific cell probabilities within each *m*‐array as a function of annual recovery and survival probabilities (Equation 2). Note, cells right (or above) of the main diagonal contain data while cells left (or below) the main diagonal $\left(i>j\right)$ are zeros.

(2) $$\begin{array}{c} \begin{array}{l} \alpha_{i,j}=\left\{\begin{array}{c} f_{\mathrm{HY},i},i=j\\ S_{\mathrm{HY},i}f_{\mathrm{AHY},i},i+1=j\\ S_{\mathrm{HY},i}\left(\prod_{k=i+1}^{k=j-1}S_{\mathrm{AHY},k}\right)f_{\mathrm{AHY},j},i+1<j<Y+1\\ 1-\Sigma_{k=1}^{k=Y}\alpha_{i,k},j=Y+1\\ 0,i>j \end{array}\right\}\;\\ \beta_{i,j}=\left\{\begin{array}{l} f_{\mathrm{AHY},i},i=j\\ \left(\prod_{k=i}^{k=j-1}S_{\mathrm{AHY},k}\right)f_{\mathrm{AHY},j},i+1<j<Y+1\;\\ 1-\Sigma_{k=1}^{k=Y}\beta_{i,k},j=Y+1\\ 0,i>j \end{array}\right\} \end{array} \end{array}$$

We used Equations 1 and 2 when fitting discrete‐time models (harvest recovery and survival) and hazard rate models (harvest recovery and unobserved mortality).

### 2.1 Discrete‐Time Model

We estimated age‐specific annual recovery and survival probabilities on the logit scale as the sum of hierarchical mean parameters (*μ*) and random effects (*ε*_*y*_) drawn from bivariate normal distributions (Equation 3): hereon, we remove the age subscripts from equations as these equations are the same for both age classes.

(3) $$\begin{array}{c} \left[\begin{array}{l} \varepsilon_{f,y}\\ \varepsilon_{S,y} \end{array}\right]\sim \text{Normal}\left(\left[\begin{array}{l} 0\\ 0 \end{array}\right],\Sigma \right)\\ \text{logit}\left(f_{y}\right)=\mu_{f}+\varepsilon_{f,y}\\ \text{logit}\left(S_{y}\right)=\mu_{S}+\varepsilon_{S,y} \end{array}\;$$

For the hierarchical means, we used Uniform prior distributions while restricting these priors to a narrower range than the parameter space of 0 and 1 per Equation 4 of Deane et al. (2023).
(4)

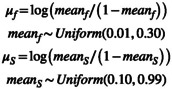




For variance–covariance matrices (**Σ**), we used Gamma(1,1) prior distributions for the standard deviations per Riecke et al. (2024) and a vague prior for correlation (Equation 5).
(5)

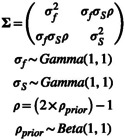




### 2.2 Hazard Rate Model

Equations (3), (4), (5) correspond to estimating annual recovery and survival as discrete time parameters. Here, we also fit hazard rate formulations of tag‐recovery models (Riecke et al. 2024). We estimated hazard rates (*h*) for cause‐specific mortalities as random effects drawn from bivariate normal distributions with hierarchical means and variance–covariance matrices. The two cause‐specific mortalities we modeled were observed harvest (*κ*) (i.e., recovery probability) and unobserved mortality (*η*) as we did not account for imperfect tag reporting and crippling loss. For the variance–covariance matrices, we use Equation (5) except that the standard deviations for unobserved mortality replace the standard deviations for survival. From observed harvest and unobserved mortality hazard rates, we derived annual recovery probability, unobserved mortality, and survival (Equation (6)): Note, *κ*_*y*_ in Equation (6) corresponds to *f*_*y*_ in Equations (2) and (3).
(6)

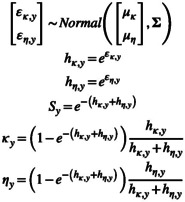




We drew hierarchical means from normal distributions that were modestly informative (Equation 7). Generally, these prior distributions cause the prior distributions for observed mortality and unobserved mortality to be < 0.5 (Appendix S1).
(7)

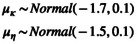




We applied hazard‐rate tag‐recovery models to the same realized datasets from Deane et al. (2023). From the known values of annual recovery and survival from Deane et al. (2023), we approximated age‐specific correlation between hazard rates by first calculating unobserved mortality probability (*u*_*y*_) as *u*_*y*_ = 1− *S*_*y*_ − *f*_*y*_, then converting these to hazard rates per Equation 8 (Ergon et al. 2018, Table 1), and then calculating correlation between hazard rates.
(8)

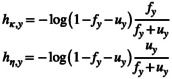




Correlation between juvenile hazard rates was 0.732 for juveniles and 0.694 for adults. The simulation of Deane et al. (2023) was based on discrete‐time parameters, not hazard rate parameters, and thus we interpret the calculated correlations between hazard rates (about 0.7) as being suggestive that the correlation between hazard rates is strongly positive.

### 2.3 Model Implementation

A common parameterization for these models is to estimate random effects for annual recovery and survival for *Y* years, but this specifies the estimation of one more survival (discrete‐time model) or natural mortality (hazard‐rate model) parameter than there are years of data. For example, our *m*‐arrays measure *Y* rows and *Y* + 1 columns from which *Y* (36) recovery parameters and *Y*–1 (35) survival parameters can be estimated (column *Y* + 1 is total individuals not recovered). In Deane et al. (2023) and many other studies, the last survival estimate (or last natural mortality parameter) is a function of the recovery parameter for year *Y* and the bivariate normal distribution from which random effects for the year are drawn. For both juveniles and adults, we now estimate *Y*–1 recovery and survival parameters (35) and correlation for *Y*–1 years. For recovery in year *Y*, we used prior distributions *f*_*HY,36*_ ~ Beta(5,95) and *f*_*AHY,36*_ ~ Beta(5,95) on the real scale (Appendix S1); these informative priors are based on the simulated parameter value and are less certain than the posterior distributions.

To better understand parameter shrinkage relative to different sample sizes and model combinations, we calculated Mean Squared Error (MSE) for the point estimates of real parameters (50% quantile). We did not calculate MSE for the full posterior distribution to reduce the influence of parameter uncertainty on our calculations. For each combination of sample size, model type, and parameter, we split MSE calculations by the years in which the true parameter value was within 1 standard deviation of the true mean or outside of 1 standard deviation of the true mean. We multiplied MSE by 10,000 so that we could present these values as whole numbers rounded to three digits. We expected MSE to be higher for real parameters outside 1 standard deviation when sample sizes were smaller due to parameter shrinkage, and we expected this pattern to be more prevalent for annual survival than annual recovery. Also, unlike Deane et al. (2023) and other correlation analyses, we now present the posterior distributions of correlations in place of point estimates, which is important given a propensity for correlations to be estimated with low certainty (Fay et al. 2022).

In Program JAGS, normal distributions expressed with variance–covariance matrices in place of precision matrices should be used with the function *dmnorm.vcov()* (Plummer 2003). We used this function when drawing random effects with Gamma(1,1) as the prior distribution for standard deviations and when we used the Uniform(0,5) prior distribution for the standard deviations in Deane et al. (2023). In contrast, the hierarchical means for our hazard‐rate models are drawn from normal distributions with a mean and a precision parameter (Equation 7) using the function *dnorm()*; precision parameters are the default expression of normal distributions in JAGS (Plummer 2003). We fit models in JAGS Version 4.3.0 (Plummer 2003) in Program R using the R packages coda (Plummer et al. 2006) and jagsUI (Kellner 2019) by running four MCMC chains for 50,000 iterations while applying a thin rate of 100 after discarding the first 10,000 values of each chain, resulting in a posterior sample of 1600 values for each parameter.

## 3 Results

**FIGURE 1:**
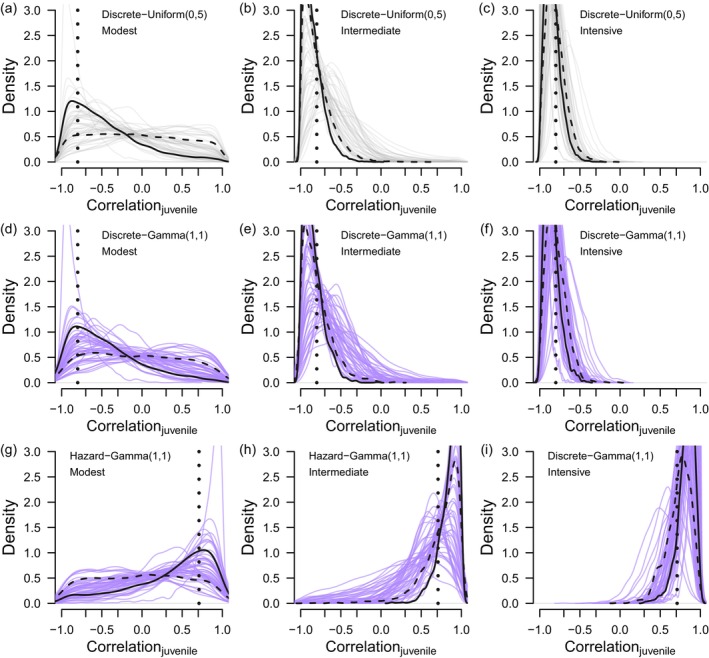
Posterior distributions of correlations between juvenile recovery and survival by model type, prior distribution for the standard deviations, and monitoring scenario: Modest, intermediate, or intensive. (a–c) are correlations between recovery and survival estimated with discrete‐time models and Uniform(0,5) priors, (d–f) are correlations between recovery and survival estimated with discrete‐time models and Gamma(1,1) priors, and (g–i) are correlations between mortality hazard rates and Gamma(1,1) priors. Each figure within each column displays 50 posterior distributions for the same 50 data realizations. Correlation for the first (solid line) and last (dashed line) data realization are highlighted. The vertical‐dotted line in the top two rows (a–f) represents true correlation (*ρ*_*R,HY*_ = −0.801) while the vertical‐dotted line in the bottom row (g–i) represents calculated correlation between hazard rates (0.732).

**FIGURE 2:**
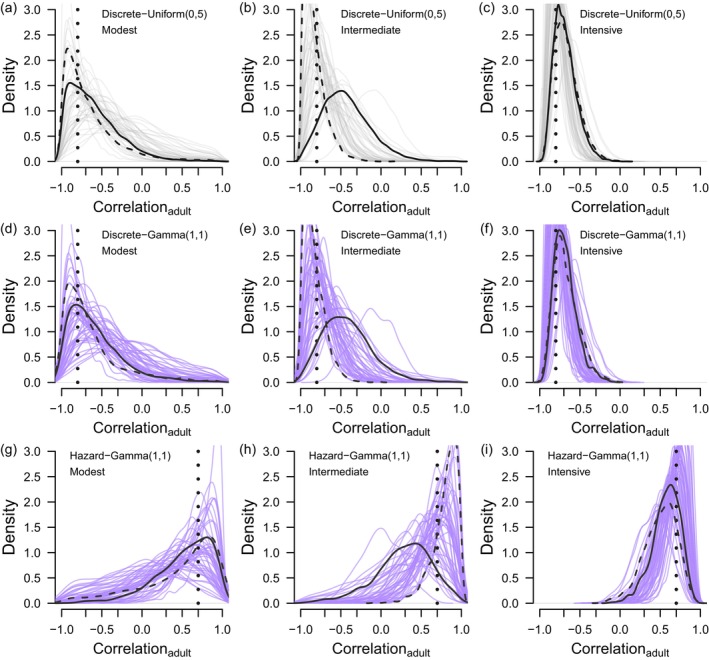
Posterior distributions of correlations between adult recovery and survival by model type, prior distribution for the standard deviations, and monitoring scenario: Modest, intermediate, or intensive. (a–c) are correlations between recovery and survival estimated with discrete‐time models and Uniform(0,5) priors, (d–f) are correlations between recovery and survival estimated with discrete‐time models and Gamma(1,1) priors, and (g–i) are correlations between mortality hazard rates and Gamma(1,1) priors. Each figure within each column displays 50 posterior distributions for the same 50 data realizations. Correlation for the first (solid line) and last (dashed line) data realization are highlighted. The vertical‐dotted line in the top two rows (a–f) represents true correlation (*ρ*_*R,AHY*_ = −0.787) while the vertical‐dotted line in the bottom row (g–i) represents calculated correlation between hazard rates (0.694).

With discrete‐time models, we found little difference between the posterior distributions of correlations estimated with Gamma(1,1) or Uniform(0,5) prior distributions for the standard deviations (Figure 1a–f, Figure 2a–f). Note, correlations estimated with Uniform(0,5) priors are comprehensively summarized our original manuscript (see Deane et al. 2023). With large cohorts of 10,000 individuals tagged annually (intensive monitoring scenario), age‐specific estimates of correlation between recovery and survival were strongly negative and generally estimated with enough certainty to not overlap 0 (Figure 1, Figure 2). When correlations estimated with Gamma(1,1) priors were summarized over all posterior distributions (50 data realizations × 1600 posterior samples), correlations estimates were strongly negative for both juveniles (${\hat{\rho }}_{\mathrm{HY}}$= − 0.841, 95% credible interval [−0.989, –0.492]) and adults (${\hat{\rho }}_{\mathrm{AHY}}$= − 0.751 [−0.928, –0.380]); both median estimates are close to the known values of these parameters (*ρ*_*R,HY*_ = −0.801, *ρ*_*R*,AHY_ = −0.787) (Deane et al. 2023). When 2000 individuals of each age class were tagged annually in the intermediate scenario, correlation estimates were less certain, and credible intervals included 0 for both age classes (${\hat{\rho }}_{\mathrm{HY}}$= − 0.746 [−0.989, −0.047]; ${\hat{\rho }}_{\mathrm{AHY}}$= − 0.708 [−0.981, 0.060]). In the modest scenario in which 250 juveniles and 800 adults were tagged annually, most posterior estimates spanned the parameter space (−1,1) and provided inconclusive inference. In this modest scenario, correlations for juveniles provided similar support for negative or positive correlation (${\hat{\rho }}_{\mathrm{HY}}$= − 0.294 [−0.965, 0.894]) whereas correlations for adults were more negative than positive (${\hat{\rho }}_{\mathrm{AHY}}$= − 0.581 [−0.979, 0.605]). Correlations between observed and unobserved mortality from hazard‐rate models were estimated with similar certainty as correlations estimated discrete‐time models (Figure 1g–i, Figure 2g–i). Age‐specific correlations between hazard rates were strongly positive when sample sizes were large, which is consistent with the values of correlation we calculated for the population we simulated (about 0.7).

**FIGURE 3:**
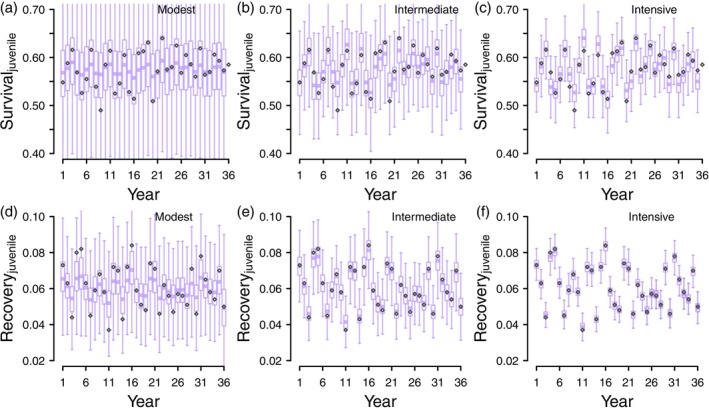
Annual estimates of juvenile survival (a–c) and recovery (d–f) from discrete‐time models when using Gamma(1,1) as the prior distribution for standard deviations. Each boxplot summarizes posterior distributions from 50 data realizations corresponding to each monitoring scenario: Modest (250 juveniles and 800 adults tagged annually), intermediate (2000 juveniles and 2000 adults tagged annually), and intensive (10,000 juveniles and 10,000 adults tagged annually). The different scales of the *y*‐axis between figures showing survival (upper panels) versus recovery (lower panels).

**FIGURE 4:**
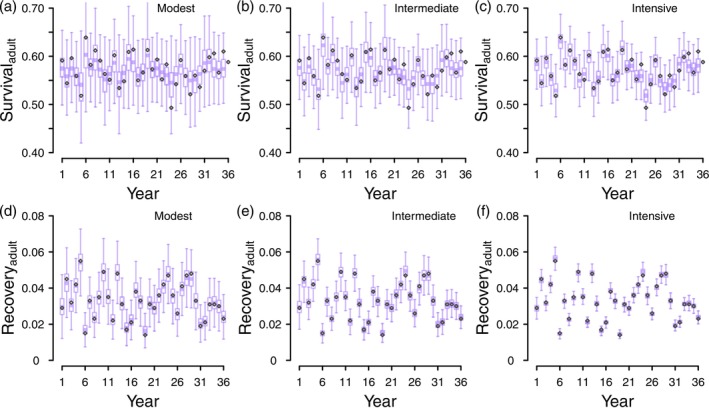
Annual estimates of adult survival (a–c) and recovery (d–f) from discrete‐time models when using Gamma(1,1) as the prior distribution for standard deviations. Each boxplot summarizes posterior distributions from 50 data realizations corresponding to each monitoring scenario: Modest (250 juveniles and 800 adults tagged annually), intermediate (2000 juveniles and 2000 adults tagged annually), and intensive (10,000 juveniles and 10,000 adults tagged annually). The different scales of the *y*‐axis between figures showing survival (upper panels) versus recovery (lower panels).

When fitting either model type while using Gamma(1,1) as the prior distribution for the standard deviations, recovery probabilities were estimated with higher certainty and less bias than survival probabilities (Figures 3 and 4, Appendix S2). In the modest scenario, juvenile recovery and survival estimates were more uncertain (Figure 3a, Figure 3d) than adult recovery and survival estimates (Figure 4a, Figure 4d) which is a predictable result given the smaller juvenile cohorts (250 juveniles tagged annually versus 800 adults). We found little difference between discrete‐time models and hazard‐rate models in terms of the bias and certainty of recovery and survival probabilities (Table 1, Figures 3 and 4, Appendix S2). Generally, recovery probabilities were estimated with high certainty such that the magnitude of 95% credible intervals did not mask the true variation of the known parameter values, though this was not true for juvenile recovery probability in the modest scenario (Figure 3d). Annual survival probabilities were less certain when compared to recovery probabilities, and it was only in the intensive scenarios for each age class that the magnitude of the 95% credible intervals did not mask the true variability of the simulated parameters (Figure 3, Figure 4). Mean squared errors (MSE) were generally at least one order of magnitude lower for recovery probabilities than survival probabilities, further demonstrating that recovery probabilities are estimated with more certainty than survival probabilities (Table 1). The difference in MSE between years outside of 1 SD of the mean and years within 1 SD was higher when sample sizes decreased (Table 1) and this difference was greater for survival probabilities than recovery probabilities (Table 1). These results (coupled with Figures 3 and 4) suggest that the parameter shrinkage towards the mean is most prevalent for survival probabilities estimated from small sample sizes and least prevalent for recovery probabilities estimated from large sample sizes, with there being little difference between model types (Table 1).

**TABLE 1:** Mean squared error (MSE) of median real parameter estimates relative to simulated recovery (*f*) and survival (*S*) probabilities by age class, juvenile (HY) or adult (AHY), and model type, discrete time or hazard rate. For each age and model combination, MSE is displayed for years in which the simulated real parameter was within one standard deviation of the mean parameter estimate (within 1SD) or years in which the simulated real parameter was outside one standard deviation of the mean (out 1SD). Each cell displays median and standard deviation of 1750 MSE values (35 annual point estimates × 50 data realizations).

	HY (within 1SD)	HY(out 1SD)	AHY(within 1SD)	AHY(out 1SD)
Recovery				
Discrete_Modest_	0.304 (1.082)	1.120 (1.516)	0.059 (0.196)	0.061 (0.222)
Hazard_Modest_	0.301 (1.113)	1.065 (1.508)	0.059 (0.195)	0.065 (0.220)
Discrete_Intermediate_	0.106 (0.314)	0.114 (0.349)	0.019 (0.071)	0.024 (0.086)
Hazard_Intermediate_	0.106 (0.315)	0.116 (0.350)	0.020 (0.070)	0.023 (0.086)
Discrete_Intensive_	0.024 (0.074)	0.026 (0.094)	0.006 (0.017)	0.006 (0.032)
Hazard_Intensive_	0.024 (0.074)	0.025 (0.094)	0.006 (0.017)	0.007 (0.031)
Survival				
Discrete_Modest_	8.777 (28.705)	19.237 (37.555)	2.085 (5.712)	8.503 (11.556)
Hazard_Modest_	8.388 (22.901)	19.659 (38.968)	1.927 (5.175)	7.548 (10.183)
Discrete_Intermediate_	3.328 (10.178)	5.724 (13.780)	1.507 (0.071)	4.776 (7.795)
Hazard_Intermediate_	3.015 (9.462)	5.95 (14.605)	1.511 (0.070)	4.815 (7.538)
Discrete_Intensive_	2.218 (5.529)	1.800 (6.578)	0.940 (0.017)	1.660 (4.349)
Hazard_Intensive_	2.161 (5.419)	1.752 (6.684)	0.941 (0.017)	1.733 (4.197)

*Note:* We multiplied MSE values by 10,000.

## 4 Discussion

When using Gamma(1,1) prior distributions for the standard deviations, which we recommend over the Gamma parameterizations we used in Deane et al. (2023) due to the error identified by Riecke et al. (2024), we found tag‐recovery datasets with sample sizes like the modest and intermediate scenarios are inadequate for estimating negative correlations between annual recovery and survival with meaningful certainty, which is a primary conclusion of Deane et al. (2023). This conclusion is important because sample sizes for most waterfowl species in North America are more comparable to the datasets used in the modest or intermediate scenarios than the intensive monitoring scenario. We included results from hazard‐rate models because these models more realistically account for competing and continuous cause‐specific mortalities (Ergon et al. 2018) than the discrete‐time models we used in Deane et al. (2023). In addition, discrete‐time formulations of tag‐recovery models parameterized with independent logit links do not constrain annual mortalities and survival to < 1, which can result in these annual probabilities summing > 1 when sample sizes are modest or when fitting these models to data from longer‐lived species like geese (unpublished results). While hazard‐rate models are advantageous over discrete‐time models for these reasons, the real parameters estimated by these models indicate that the same data limitations apply to hazard‐rate models as discrete‐time models.

A consistent pattern in our results is more certain estimates of annual recovery than annual survival, which suggests that the uncertainty associated with estimating annual survival is more limiting to inference than the uncertainty of annual recovery. While discrete‐time models estimate annual survival as random effects, hazard‐rate models estimate unobserved mortality hazard rates as random effects such that annual survival is a derived parameter. Furthermore, increasing shrinkage of random effects as data become more limiting is expected (Royle and Link 2002) and both mean squared error (Table 1) and summary figures (Figures 3 and 4) indicate annual recovery estimates are less prone to shrinkage than annual survival (or apparent shrinkage of annual survival in the case of hazard rate models). Regardless of whether survival or unobserved mortality hazard rate is the estimated parameter, the consistency in results between both model types highlights that harvest recoveries directly inform one cause‐specific mortality type, but without independent information about survival (e.g., recaptures or resightings) or another mortality type, either survival (discrete‐time models) or unobserved mortality (hazard‐rate models) will be estimated with more uncertainty than recovery probability (or observed harvest hazard rate). These results suggest a fundamental challenge to estimating correlation between directly informed parameters (*f*_*y*_ [discrete‐time models] or *h*_*κ,y*_ [hazard‐rate models]) and indirectly informed parameters (*S*_*y*_ [discrete‐time models] or *h*_*η,y*_ [hazard‐rate models]) is higher uncertainty of the indirectly estimated parameters. Beyond correlations, our results indicate more potential for underestimating the variability of survival than harvest recovery from empirical datasets with sample sizes similar to our modest and intermediate scenarios.

Our original paper (Deane et al. 2023), the comment by Riecke et al. (2024), and this response are part of an important process of (1) identifying shortcomings in existing methods (e.g., Ergon et al. 2018) and (2) understanding if a study has adequate power to address a question of interest (e.g., Johnson et al. 2015). Simulation studies or power analyses are often limited in that more scenarios could be assessed. For example, we did not simulate a new population from hazard rates, which kept us from assessing the bias of correlations between hazard rates. Similarly, we could have assessed tag‐recovery models parameterized with Seber *r* recovery parameters (Sedinger et al. 2010), models fit to both live recapture and tag‐recovery data (Koons et al. 2014), and different vital rate scenarios (duck‐like versus goose‐like). Alternately, we could have assessed more formidable assumptions like using a single correlation parameter to describe the relationship between two demographic parameters over multi‐decadal study periods, like harvest and survival, when the underlying relationships may change. Fay et al. (2022) note that correlation analyses are often challenged by high uncertainty and underestimation of the magnitude, such that the direction of correlation parameters may be the only interpretable attribute of these parameters. We suggest this cautious approach to interpretation is relevant to correlation analyses using tag‐recovery data, and in some cases, these correlation estimates may be so imprecise that it may be best to forego interpretation. Our updated and expanded results that incorporate the correction suggested by Riecke et al. (2024) reinforce our previous conclusions about the inadequacy of correlations for inference when estimated from datasets with sample sizes similar to our modest and intermediate scenarios.

## Author Contributions

**Cody E. Deane:** formal analysis (lead), writing – original draft (lead), writing – review and editing (equal). **Lindsay G. Carlson:** writing – review and editing (equal). **Curry J. Cunningham:** writing – review and editing (equal). **Pat Doak:** writing – review and editing (equal). **Knut Kielland:** writing – review and editing (equal). **Greg A. Breed:** formal analysis (lead), writing – original draft (lead), writing – review and editing (equal).

## Conflicts of Interest

The authors declare no conflicts of interest.

## Supporting information


**Data S1.** Supporting Information.

## Data Availability

The realized datasets from the simulation study in Deane et al. (2023) that are used in this analysis are available with new R scripts at https://datadryad.org/stash/share/GkoS_z‐_nnSVY3by‐gbv8_fyYrNFS4u5FsE_KdBxGgI.
