# Supplementary material for: Accurately Estimating Correlations Between Demographic Parameters: A Response to Riecke Et al. (2024)
Source: Ecol Evol. 2025 Feb 24;15(2):e71004. doi: 10.1002/ece3.71004 (PMC11850757; doi:10.1002/ece3.71004)
Supplement: Supplementary file 1 — Data S1. Supporting Information. [file ECE3-15-e71004-s001.docx]

**Deane, C.E., L.G. Carlson, C.J. Cunningham, P. Doak, K. Kielland, and G.A. Breed.**

**Accurately estimating correlations between demographic parameters: a response to Riecke et al. (2024)**

**Ecology and Evolution – Appendix S1**

Prior distributions of real parameters while using the prior distributions *μ_κ_* ~ *Normal(–1.7,0.1*) and *μ_η_* ~ *Normal(–1.5,0.1)*. The priors we chose result in three real parameters having median values below 0.5, but the distributions of these prior distributions still covered the available parameter space (0,1). We found these priors restricted parameter values to range of values that did not require Program JAGS rounding to *-Inf* or *Inf* (depending on the parameter). Our preliminary modeling found the prior distributions *μ_κ_* ~ *Normal(–1.7,0.5*) and *μ_η_* ~ *Normal(–1.5,0.5)* pushed annual survival to a median of about 0.6 and the medians of observed and unobserved mortality closer to 0.


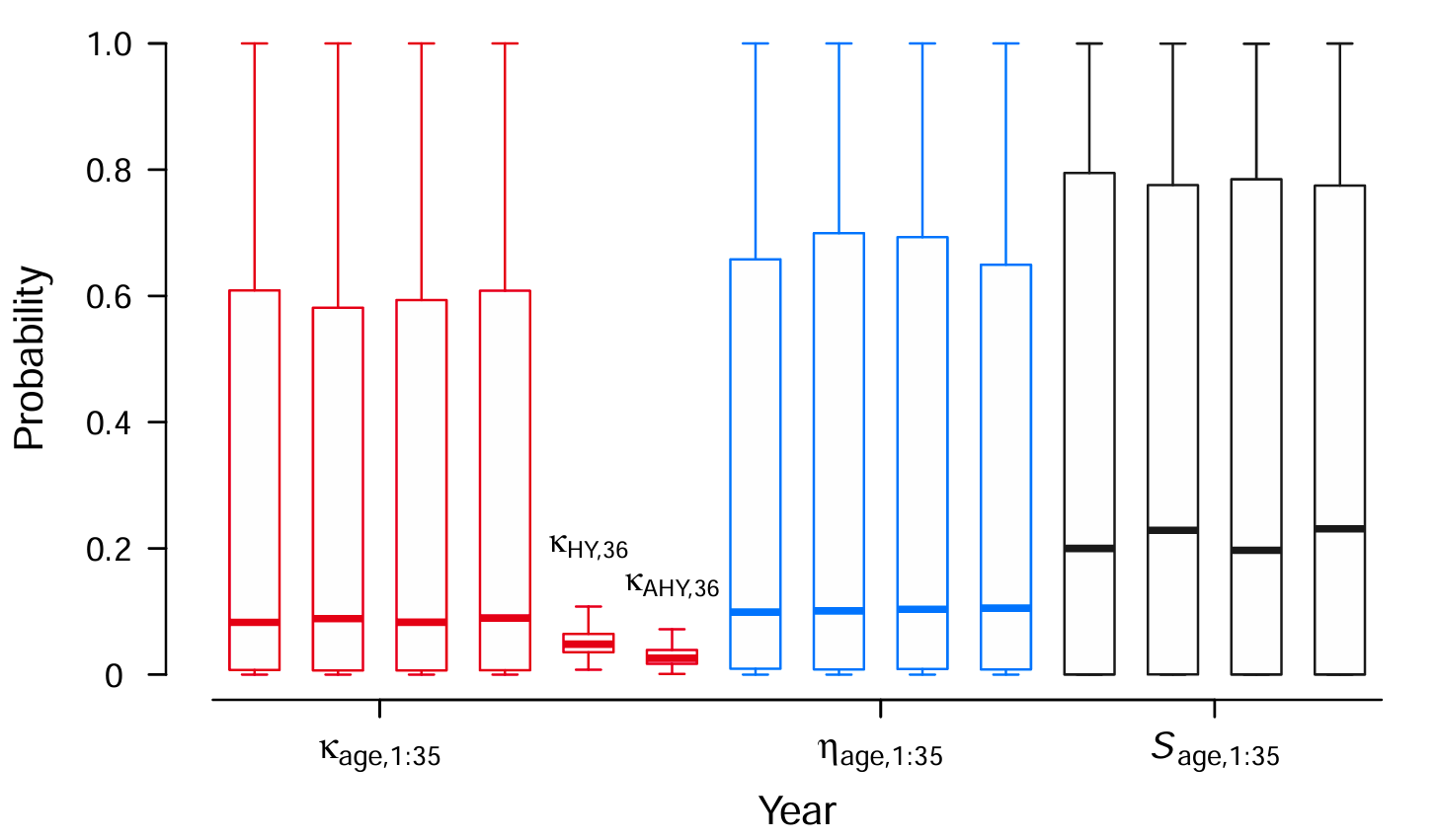


**Figure S1–1.** Boxplots of prior distributions for real parameters observed mortality (*κ*), unobserved mortality (*η*), and survival (*S*) for years 1–35 in red, blue, and black, respectively. The prior distributions we used for the last recovery parameter in year 36 (for both the discrete-time and hazard-rate models) are labeled *κ_HY,36_* and *κ_AHY,36_*.

**Deane, C.E., L.G. Carlson, C.J. Cunningham, P. Doak, K. Kielland, and G.A. Breed.**

**Accurately estimating correlations between demographic parameters: a response to Riecke et al. (2024)**

**Ecology and Evolution – Appendix S2**


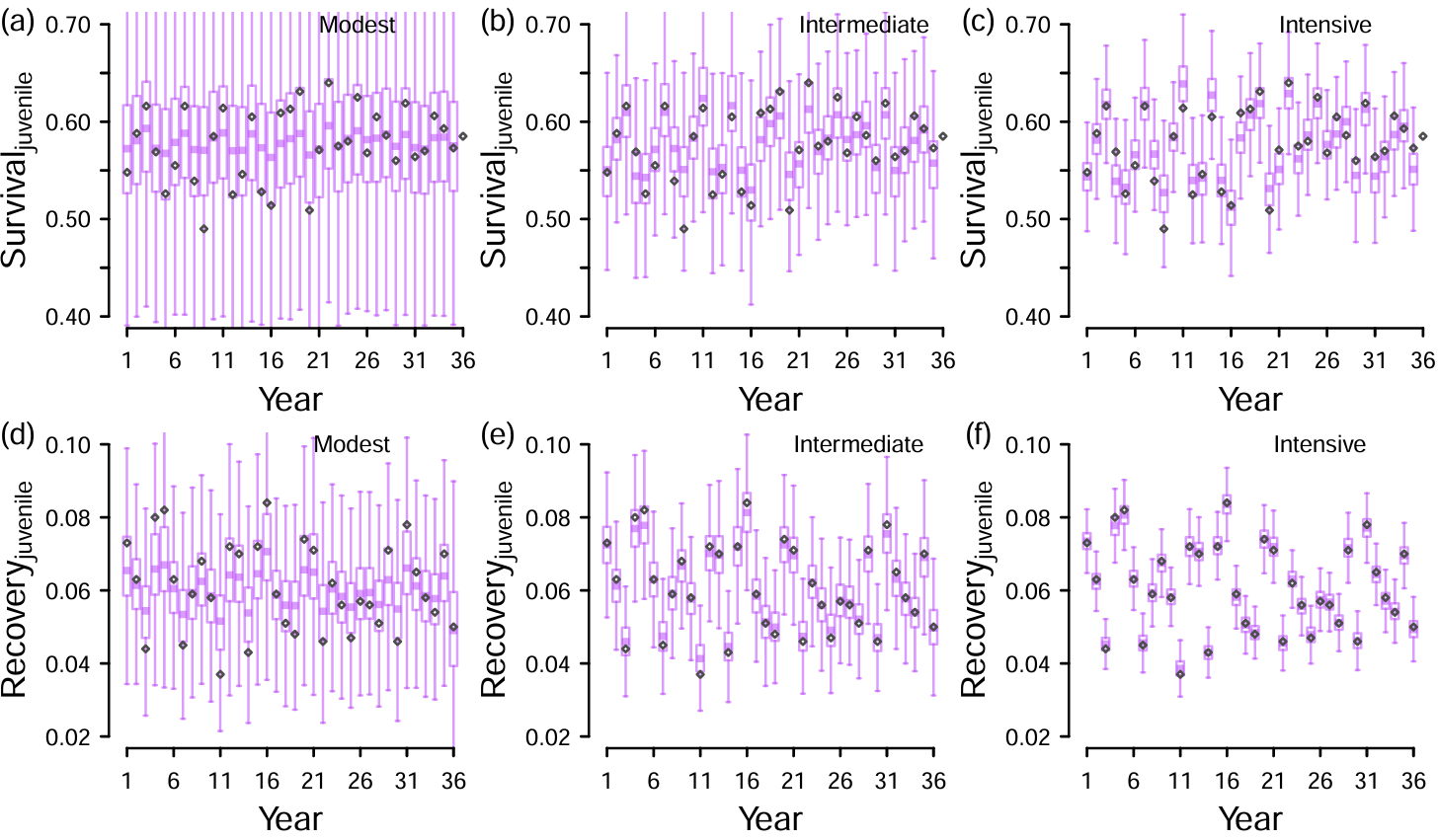


**Figure S2–1.** Annual estimates of juvenile survival (Fig. a–c) and recovery (Fig. d–f) hazard-rate models when using Gamma(1,1) as the prior distribution for standard deviations. Each boxplot summarizes posterior estimates from 50 data realizations corresponding to each monitoring scenario: modest (250 juveniles and 800 adults tagged annually), intermediate (2000 juveniles and 2000 adults tagged annually), and intensive (10,000 juveniles and 10,000 adults tagged annually). Note this figure is qualitatively identical to Figure 3.


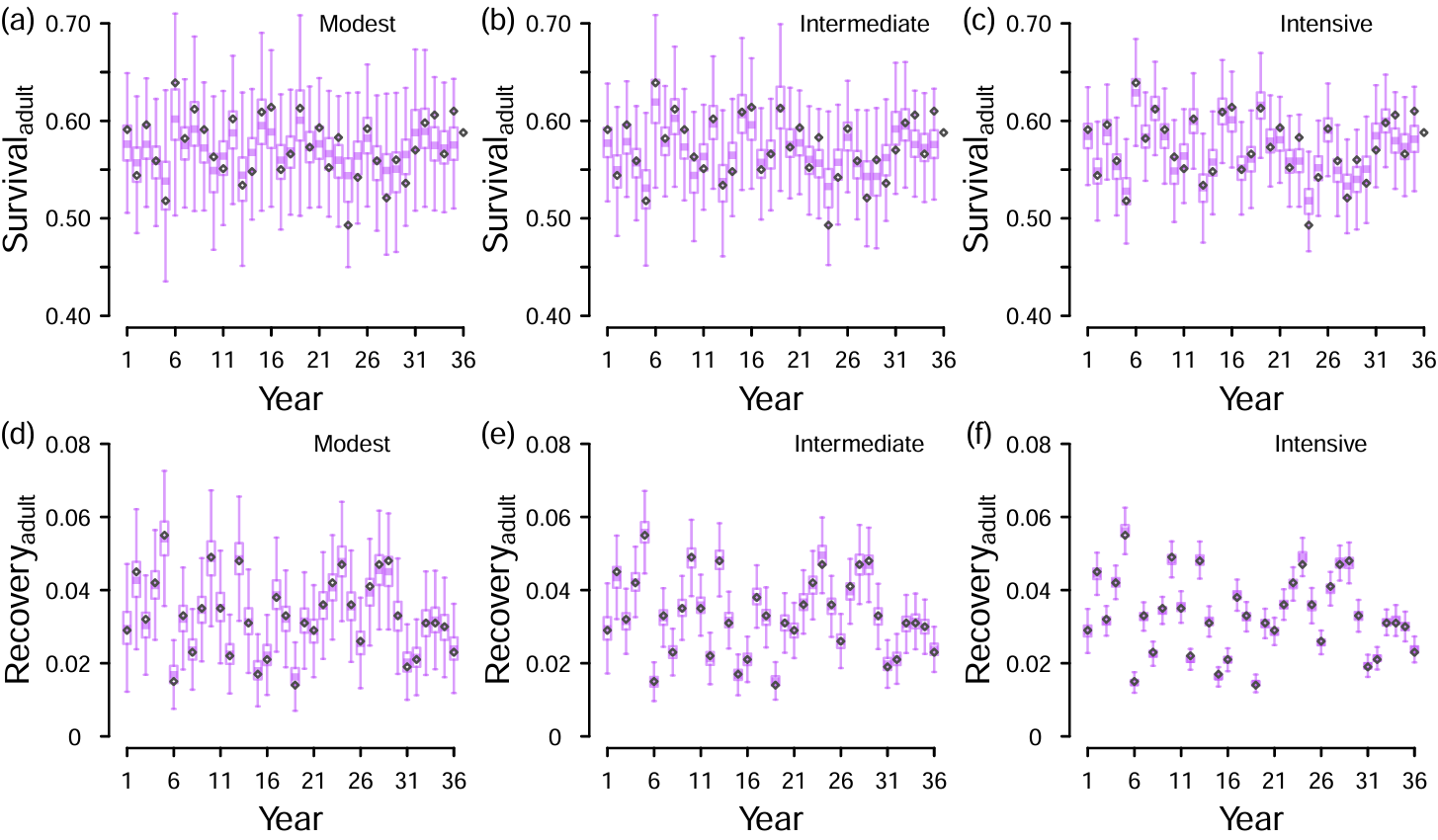


**Figure S2–2.** Annual estimates of adult survival (Fig. a–c) and recovery (Fig. d–f) hazard-rate models when using Gamma(1,1) as the prior distribution for standard deviations. Each boxplot summarizes posterior estimates from 50 data realizations corresponding to each monitoring scenario: modest (250 juveniles and 800 adults tagged annually), intermediate (2000 juveniles and 2000 adults tagged annually), and intensive (10,000 juveniles and 10,000 adults tagged annually). Note this figure is qualitatively identical to Figure 4.
